# Quantitative text feature analysis of autobiographical interview data: prediction of episodic details, semantic details and temporal discounting

**DOI:** 10.1038/s41598-017-14433-6

**Published:** 2017-11-08

**Authors:** J. Peters, A. Wiehler, U. Bromberg

**Affiliations:** 1 Department of Psychology, Biological Psychology, University of Cologne, Cologne, Germany; 2 Department of Systems Neuroscience, University Medical Center Hamburg-Eppendorf, Hamburg, Germany; 3 Motivation, Brain and Behavior lab, Institut du Cerveau et de la Moelle épinière, Paris, France; 4 Inserm Unit 1127, CNRS Unit 7225, Université Pierre et Marie Curie (UPMC - Paris 6), Paris, France

## Abstract

Autobiographical memory and episodic future thinking (i.e. the capacity to project oneself into an imaginary future) are typically assessed using the Autobiographical Interview (AI). In the AI, subjects are provided with verbal cues (e.g. “your wedding day”) and are asked to freely recall (or imagine) the cued past (or future) event. Narratives are recorded, transcribed and analyzed using an established manual scoring procedure (Levine *et al*., 2002). Here we applied automatic text feature extraction methods to a relatively large (n = 86) set of AI data. In a first proof-of-concept approach, we used regression models to predict internal (episodic) and semantic detail sum scores from low-level linguistic features. Across a range of different regression methods, prediction accuracy averaged at about 0.5 standard deviations. Given the known association of episodic future thinking with temporal discounting behavior, i.e. the preference for smaller-sooner over larger-later rewards, we also ran models predicting temporal discounting directly from linguistic features of AI narratives. Here, prediction accuracy was much lower, but involved the same text feature components as prediction of internal (episodic) details. Our findings highlight the potential feasibility of using tools from quantitative text analysis to analyze AI datasets, and we discuss potential future applications of this approach.

## Introduction

Autobiographical memory (AM) is central to our personal identity, and changes in this process characterize developmental phases as well as effects of neurological and psychiatric disorders. Recent findings have illustrated striking similarities in the neural systems supporting episodic memory and the capacity to mentally project oneself into the future (episodic future thinking, EFT)^1^. For example, AM and EFT have been shown to be affected (albeit to partly different degrees) in hippocampal amnesia^2,3^, Alzheimer’s Disease^4,5^, normal aging^6^ and traumatic brain injury^7^, highlighting the close association between memory and future event construction^1,8^.

In addition, EFT impacts directly on other cognitive functions. For example, it has been speculated that EFT may facilitate future-oriented choice behavior, i.e. behavior that is advantageous only in the long-run^9^. One way to assess this type of behavior is via temporal discounting. In these paradigms, the relative preference for smaller-sooner rewards over larger-but-later rewards is measured^10,11^. A stronger preference for smaller-sooner rewards is taken as a measure of impulsivity, whereas a stronger preference for larger-later rewards is taken to reflect more future-oriented preferences. Boyer (2008) originally speculated that the ability to use EFT to project oneself into the future may help humans to override a natural tendency to make impulsive and short-sighted decisions, i.e. it may reduce the degree of temporal discounting. Recent years have brought forth increasing empirical support for the idea that EFT may, under certain conditions, modulate temporal discounting in this manner^12–18^. These interactions are particularly relevant for psychiatry, since steep discounting is a reliable behavioral marker for a range of disorders of impulse control, including substance abuse and pathological gambling^19^. Therefore, understanding mechanisms of how temporal discounting can be reduced is of high clinical relevance.

Inter-individual differences in AM and EFT are typically assessed using variations of the autobiographical interview (AI)^20^. The AI procedure involves exposing participants to a number of cues referring to future or past events, with the instruction to vividly recall (AM) or imagine (EFT) these events. Participants verbally elaborate on their memories (AM) or imaginations (EFT) and these narratives are recorded and transcribed. Transcripts are then manually scored using established procedures designed to dissociate e.g. episodic from semantic content^20^. Thus, typical outcome measures of an AI study include sum scores of the number of *episodic details* (often termed *internal details*, as they pertain directly to the central event in question), sum scores for *external details* (episodic details not pertaining to the event in question) and sum scores for *semantic details* (non-episodic information). At present, the AI can arguably be described as the “gold standard” in measuring AM and EFT^1,21^, as it is widely used and AI scores typically show high inter-rater reliability. There are, however, a few shortcomings of the procedure.

First, the multi-step procedure (interview, transcription, scoring) is very time consuming, and this might discourage researchers from using the AI in studies with time constraints. Second, the manual scoring procedure is subjective. For this reason, typically multiple independent raters score at least a subset of the data, in order to ensure the reliability of the rating procedure. Finally, the rich and oftentimes long narratives that participants produce during an AI testing session contain a lot of linguistic information that could in principle be analyzed in a largely automatic fashion. However, by focusing mainly on manual scoring and the resulting sum scores for different detail categories (e.g. internal, semantic, external, see above), it is possible that potentially interesting information is ignored. For example, information regarding emotional valence, word concreteness, sentence length and the proportion of specific word types (e.g. adjectives, verbs) are typically not considered directly when internal details are scored. The present study provides a first step towards a more automatic and quantitative analysis of linguistic content in AI data by extracting low-level linguistic features from AI narratives in a largely automatic fashion.

We re-analyzed a large set (n = 86) of previously published AI data^22,23^ using automatic extraction of low-level text features. Text features were computed both manually by cross-referencing words with publicly available linguistic data bases, and using commercially-available quantitative text analysis software, the *Linguistic Inquiry and Word Count* (LIWC) package^24^. Note that one previous study applied the LIWC to autobiographical memory narratives^25^, but that study focused on differences in emotional content between younger and older adults. In addition, two earlier studies examine the use of past-tense verbs in AI tasks in neurodegenerative disorders^26^ and temporal lobe epilepsy^27^, but did not explore additional text features or comprehensively analyzed detail sum score prediction. The first aim of this report is an initial proof-of-concept. We aimed to assess how well AI based internal and semantic detail sum scores can be predicted from low-level linguistic features using statistical methods with a combination of dimensionality reduction and regression techniques with out-of-sample prediction (cross-validation). We then also explored whether different features are associated with e.g. internal vs. semantic details ratings. In the light of the known associations between EFT and temporal discounting (see above), we also used the same regression models to directly predict temporal discounting behavior from linguistic features of AI narratives.

## Methods

### Participants

We re-analyzed autobiographical interview (AI) data from two datasets. The first dataset (dataset 1) comprised interview data from n = 46 adolescents (age range: 12–16, 23 male). The second data set (dataset 2) comprised data from n = 20 pathological gamblers (mean age [range]: 32.9 [19–59], 19 male), and from n = 20 healthy control participants (mean age [range]: 32.55 [18–58], 19 male). All subjects provided informed written consent prior to participation. For minor participants, the parent or legal guardian provided written consent. All procedures were approved by the local ethics committee (Hamburg Board of Physicians) and all methods were conducted in accordance with the guidelines and regulations of this committee.

### Autobiographical Interview

Data were acquired using a modified version of the Autobiographical Interview (AI) Interviews were conducted by U.B. (dataset 1) and A.W. (dataset 2) using a standardized protocol. For dataset 1, each participant was first instructed to report 12 personal episodic events (3 events within the next 6 months, 3 events during the following school year, 3 events within the last 6 months and 3 events during the previous school year) from 3 different settings: 4 events related to family life, 4 events related to school life and 4 events related to their spare time activities). For dataset 2, each participant was instructed to report 5 personal episodic events that happened one year ago, and 5 personal episodic events that could happen one year from now. For further details on the cue selection procedure, please refer to the original publications^22,23^.

Verbal cues referring to each event were then presented to each participant, and they were given 3 min to freely elaborate on the respective past or future event. Following this, a standard follow-up question was asked depending on what had been told already (“Can you tell me any more about where and when the event is taking place, who is there, how you feel and what you are thinking?”). Verbal reports were digitally recorded, transcribed, and then scored according to the original AI manual^20,22,23^.

Scoring involved the manual classification of each reported piece of information (detail) into one of several content categories. Details were scored as *episodic internal details* if they contained episodic information regarding the cued event, as *episodic external details* if they referred to episodic information regarding some other non-cued event, and as *semantic details* if they referred to non-episodic factual information. Following the original manual^20^ five subcategories of internal details were differentiated: event details, time details, place details, perceptual details and emotion/thought details. For each participant, a sum score for internal and external episodic details was computed as the sum of the details across these categories.

### Temporal discounting

Temporal discounting refers to the reward devaluation that typically occurs with increasing delay. All subjects completed a simple short and adaptive discounting task^28^ that involved repeated choices between a smaller-sooner reward of 20€ available now and larger-but-later rewards available only after some delay (2, 7, 14, 30, 90, 180 days). The procedure was adaptive such that the reward amount of the larger-later reward was increased following two successive choices of the smaller-sooner reward, and decreased following two successive choices of the larger-later reward. Choice data were then fitted with a standard hyperbolic discounting function of the form $SV=A/(1+k\times D)$. Here, *SV* is the subjective discounted value of the reward, *A* is the objective reward amount, *D* is the delay (measure in days), and *k* is a subject-specific discounting function, where greater values reflect steeper discounting and thus more impulsive preferences. Fitting was performed using maximum likelihood techniques implemented in Matlab © version R2013a (The Mathworks). Details of the procedure are given elsewhere^29^. As the resulting single-subject k-parameters are not normally distributed, we applied a square-root transformation prior to analyses^29,30^.

### Text feature extraction

Computation of text features proceeded in a purely data-driven manner. That is, we did not have a priori assumptions about which low-level text features might be informative regarding AI details sum scores. Rather, we simply applied two complementary methods to extract a large number of low-level linguistic features from the narratives. Since we used regression approaches suitable for collinear data (see below), high correlations between some of the variables are not problematic per se.

First, we used custom in-lab Matlab © procedures to cross-reference words with publicly available linguistic databases (‘manual feature extraction’). Second, we used commercially available text analysis software (‘Linguistic Inquiry and Word Count’, LIWC)^31^ to obtain additional text features. The two approaches are described in detail in the following.

#### Manual text feature extraction

**Figure 1:**
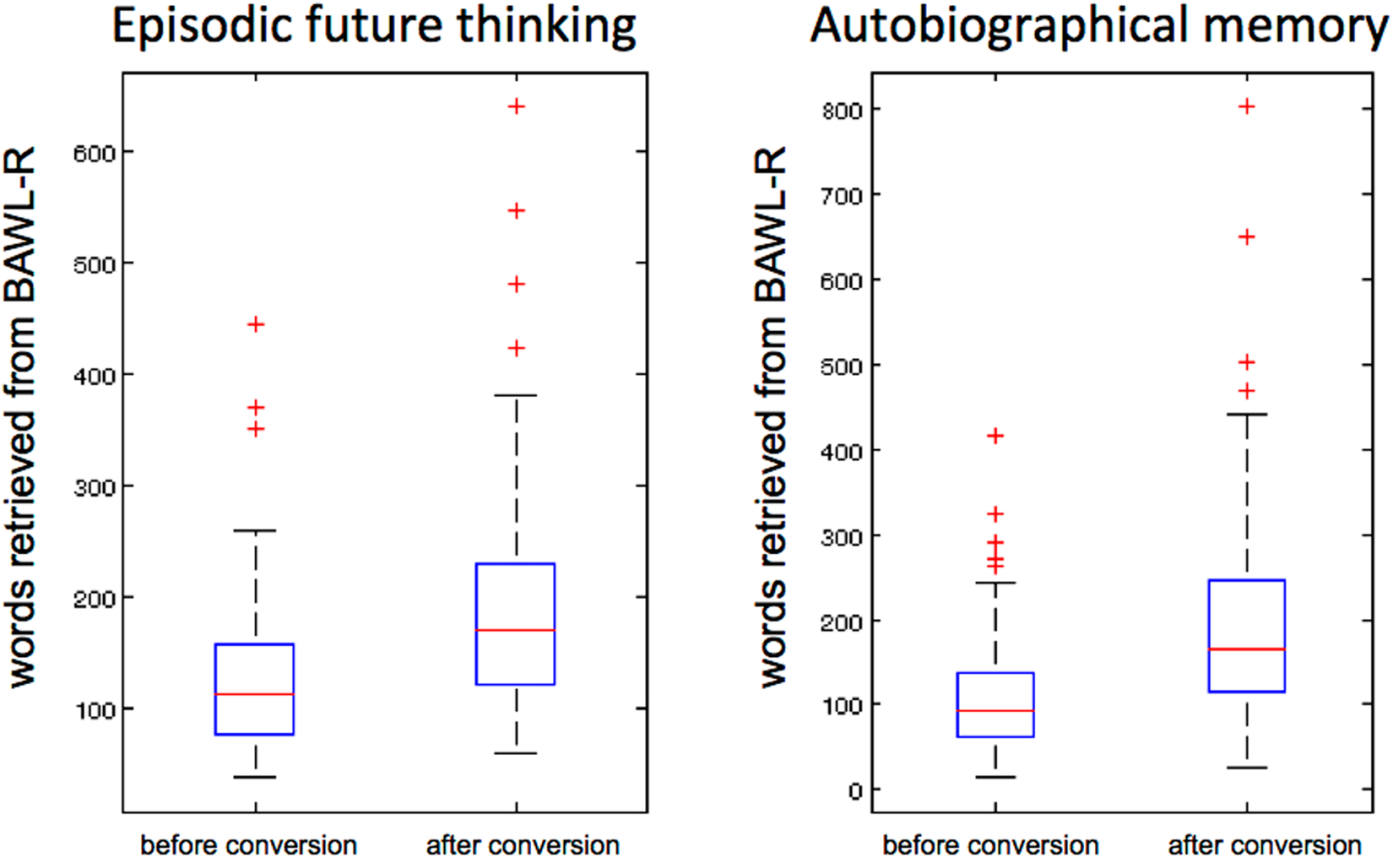
Baseform conversion substantially increased the number of words successfully cross-referenced with the BAWL-R database^36^ by around 50% for both EFT (left) and AM (right) conditions.

Transcripts of narratives were first pre-processed to extract potentially relevant text features. The data from each subject were read into Matlab © and separated according to the two experimental conditions (EFT, AM). Then, individual words and sentences were extracted from the narratives. Words were then further analyzed using Webservices provided by the “Projekt Deutscher Wortschatz” (http://wortschatz.uni-leipzig.de), which is part of the Leipzig Corpora Collection. Each word was converted to base form and classified (noun/adjective/verb/other) using the “baseform” webservice (http://wortschatz.uni-leipzig.de/axis/servlet/ServiceOverviewServlet). For an input of e.g. *freundlichste* (*nicest*), this service returns both the baseform *freundlich* (*nice*) and the classification (*adjective*). This allowed us to calculate the proportion of nouns, verbs and adjectives separately for each condition. Next, we cross-referenced words (both pre- and post- baseform conversion) with the Berlin Affective Word List – Reloaded (BAWL-R) (Vo *et al*., 2009). The BAWL-R is a database that contains ≈3000 German words (≈2100 nouns, ≈500 verbs, ≈290 adjectives). It provides normative ratings for the dimensions valence (−3 [*very negative*] through 0 [*neutral*] to +3 [*very positive*]), imageability (1 [*hardly imageable*] through 7 [*highly imageable*]), and emotional arousal (1 [*low arousal*] through 5 [*high arousal*]) as well as a measure of word frequency (frequency/million). Figure 1 illustrates that baseform conversion increased the number of words that could be successfully cross-referenced with the BAWL-R database by around 50% in both experimental conditions. Based on the BAWL-R data, we calculated mean and variance of valence, arousal and imageability ratings for each subject and condition. Note that due to the highly skewed distribution of word frequencies, we used the median rather than the mean as a summary measure for each subject and condition. Based on these data, 19 predictors per experimental condition were obtained for each participant. The features are listed and explained in Table 1.

**Table 1:** Labels and descriptions of manually-derived predictor variables (see ‘manual feature extraction’ section in the methods section).

Label	Description
n_words	total number of words per condition (across all cues)
n_sentences	total number of sentences per condtion (across all cues)
total_classified_words	total number of words successfully classified as verb/adjective/noun
n_adjectives	total number of words classified as adjectives
n_verbs	total number of words classified as verbs
n_nouns	total number of words classified as nouns
p_adjectives	n_adjectives/n_words
p_verbs	n_verbs/n_words
p_nouns	n_nouns/n_words
n_bawl	total number of words successfully cross-referenced with BAWL-R database post-baseform conversion
n_bawl_pre	total number of words successfully cross-referenced with BAWL-R database pre-baseform conversion
wps	words per sentence: n_words/n_sentences
m_emo	mean BAWL-R emotion rating
m_arousal	mean BAWL-R arousal rating
m_image	mean BAWL-R imageability rating
v_emo	variance BAWL-R emotion rating
v_arousal	variance BAWL-R arousal rating
v_image	variance BAWL-R imageability rating
med_freq	median word frequency

Each variable was computed separately for the EFT and AM conditions, yielding 38 predictors in total. BAWL-R – Berlin Affective Word List Reloaded.

Throughout the present paper, we make a simplified distinction between qualitative and quantitative manual text features. Quantitative text features refer to those text features that simply score the raw amount of verbal material (e.g. number of sentences, number of words, number of adjective etc., see Table 1). Qualitative text features, in contrast, refer more directly to word characteristics (e.g. mean word imageability, mean valence, proportion of adjectives etc., see Table 1).

#### Linguistic Inquiry and Word Count (LIWC) features

We also applied a commercially available text analysis software that is frequently used in psychological research, the Linguistic Inquiry and Word Count (LIWC). LIWC is a dictionary-based method that counts words falling into one of 64 content categories, and normalizes these counts by the total length of the texts. These categories include specific word classes (e.g. filler words, numbers, pronouns, articles) but also semantic content categories (words related to e.g. leisure, home, school, job, sports, TV) as well as cognitive-emotional categories (e.g. positive emotion, negative emotion, anxiety, affect). See results section for a complete list of LIWC content categories used in the present study. We used a validated German LIWC dictionary^31^ and separately analyzed EFT and AM data for each participant.

### Regression analyses

To assess the association between text features (i.e. manual features, LIWC features) and AI ratings we used regression techniques. Note that prediction focused on internal and semantic details, since these AI measures both showed a reasonably high correlation between AM and EFT conditions and substantial variance between subjects. In contrast, this was not the case for the external detail sum scores as well as the more specific internal details sub-categories (event, time, place emotion, perceptual), which were thus excluded from the predictive modeling.

Examination of the covariance structure of the predictor space revealed high co-linearity between some predictors (see results section). This poses a problem for standard multiple regression, as there is no unique least squares solution. We therefore applied regression techniques that can deal with collinear data^32^.

#### Principal component regression (PCR)

PCR consists of first performing a principal component analysis (PCA) on the data matrix **X**. In the next step some target vector **y** is regressed onto a subset of *n* of these components, with *n* being typically determined via cross validation. PCA is a completely data-driven approach that extracts the main axes of variation from a multi-dimensional data set. Often, a relatively small subset of these *principal directions* accounts for the majority of variability in the data, and PCA is thus an effective technique for dimensionality reduction. The resulting component scores, which are linear combinations of the original variables, are orthogonal, and PCR thus solves the problem of predictor co-linearity. PCR was performed by first computing a PCA using the Matlab function *pca*. Then a regression analysis using the resulting component scores as predictors was implemented using the Matlab functions *regress* and *pcr_sse*.

#### Partial least squares regression (PLS)

PCR uses PCA to construct new orthogonal predictor variables, but it is completely data driven – components are constructed without regard to the target data **y** that one wishes to eventually predict. Thus it is possible that information predictive of **y** ends up in later components that are excluded from the PCR. Thus, when, the ultimate goal is *prediction*, PCR may not be the optimal choice. In contrast to PCA, in PLS, components are constructed based on both **X** and **y**, that is, components are constructed such as to jointly maximize the amount of variance that is explained in **X** *and* the correlation of the resulting components with **y** ^32^. We performed PLS using the Matlab function *plsregress*, which also implements cross-validation.

### Alternative regression approaches

For comparison with PCR and PLS, and to ensure that our results are independent of the regression approach adopted, we applied two additional techniques.

#### Supervised PCR (sPCR)

Supervised PCR^33^ is a recently proposed extension of standard PCR that involves an additional variable selection step. Columns of **X** that show little correlation with **y** are excluded before the PCA step of PCR. Which predictors are excluded is determined by the inclusion threshold $\theta$. The optimal value for $\theta$ is again determined by cross-validation (see below).

#### Ridge regression

In ridge regression, the parameters of multiple linear regression are shrunk towards zero, with the degree of shrinkage being determined by the ridge tuning constant $\lambda$, which is determined by cross-validation. Shrinkage solves the problem of high variance in linear regression parameter estimates when predictors are highly correlated. We used the Matlab function *ridge*, which also implements cross-validation for the tuning of $\lambda$.

### Cross-validation

We used leave-one-out cross validation to quantify the out-of-sample prediction accuracy of all regression models, as well as to tune model hyper-parameters (e.g. the shrinkage parameter $\lambda$ in ridge regression). To this end, models were fit to the data of all but one participant. We then calculated the root mean squared cross validation error (RMSE) across subjects as $RMSE=\sqrt{\frac{{\sum }_{i=1}^{n}{({\hat{y}}_{i}-{y}_{i})}^{2}}{n}}$ where ${\hat{y}}_{i}$ and ${y}_{i}$ are the predicted and actual data for the *i*-th subject. For the case of PCR and PLS, this procedure was repeated for a range of models with different numbers of included components in order to identify models maximizing out-of-sample prediction (i.e. minimizing over-fitting the training data).

## Results

### Autobiographical memory interview data

**Figure 2:**
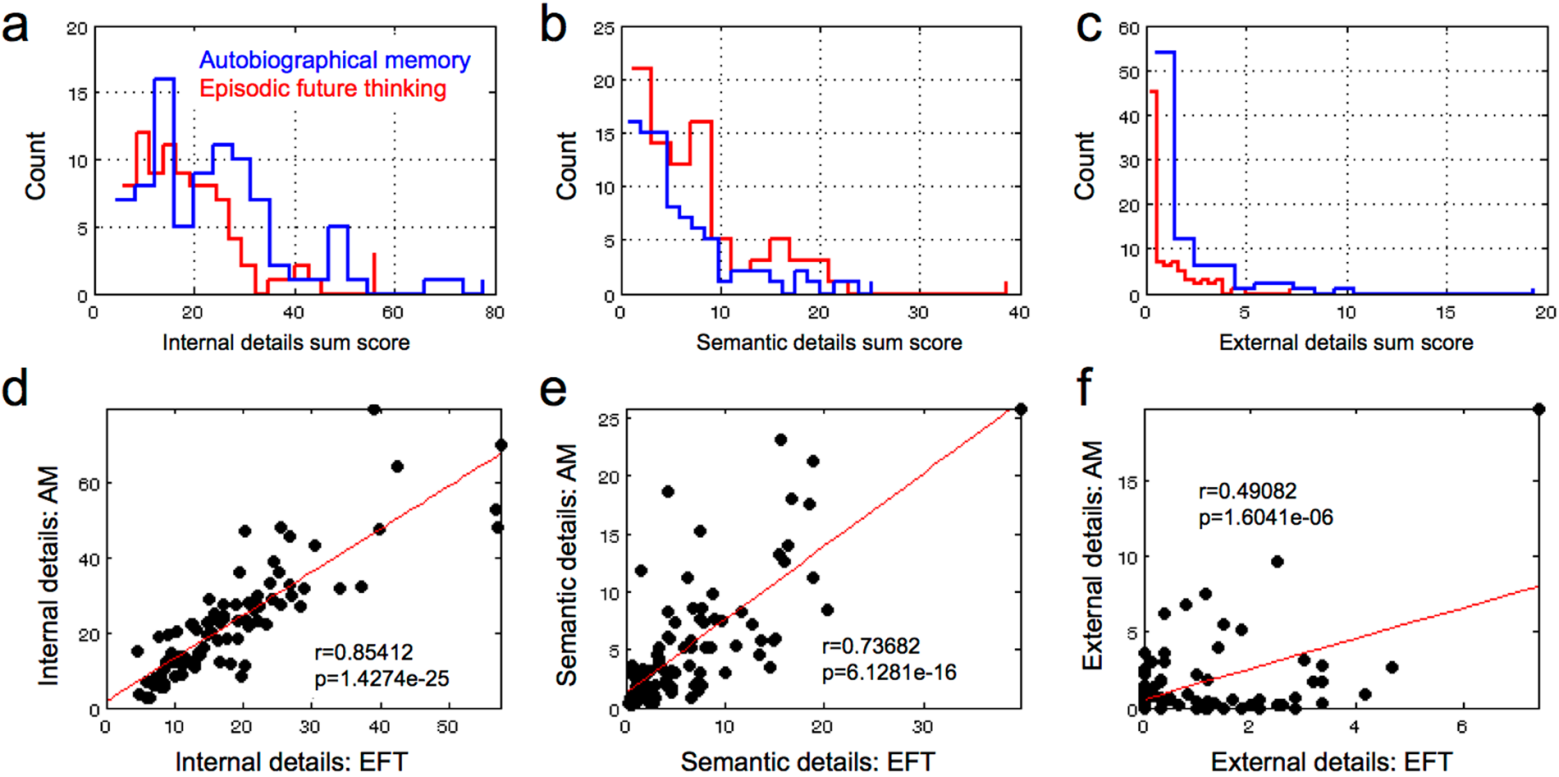
Autobiographical interview (AI) data. Distributions of internal and semantic detail sum scores for each condition (blue – autobiographical memory [AM], red – episodic future thinking [EFT]) are shown in top row, and sum score correlations between AM and EFT are shown in the bottom row. From left to right: internal episodic details, semantic details, external episodic details.

Figure 2a–c shows histograms of the distributions of ratings for internal, semantic and external details. Overall, internal details ratings (t_85_ = 5.0057, p < 0.001) and external details ratings (t_85_ = 2.236, p = 0.028) where higher for AM than EFT, whereas semantic details ratings were higher for EFT than AM (t_85_ = 2.5189, p = 0.014). All ratings were significantly correlated between conditions (Fig. 2d–f).

### Text feature data

We used two types of extracted text features (see methods section for details). First, text features were manually extracted using custom Matlab © code, in combination with research-dedicated word databases (see Methods section for details and Table 1 for an overview of manually computed features). Second, we used commercially available software (Linguistic Inquiry and Word Count, see methods section) to derive dictionary-based word counts for 64 content categories.

### Data correlation structure

**Figure 3:**
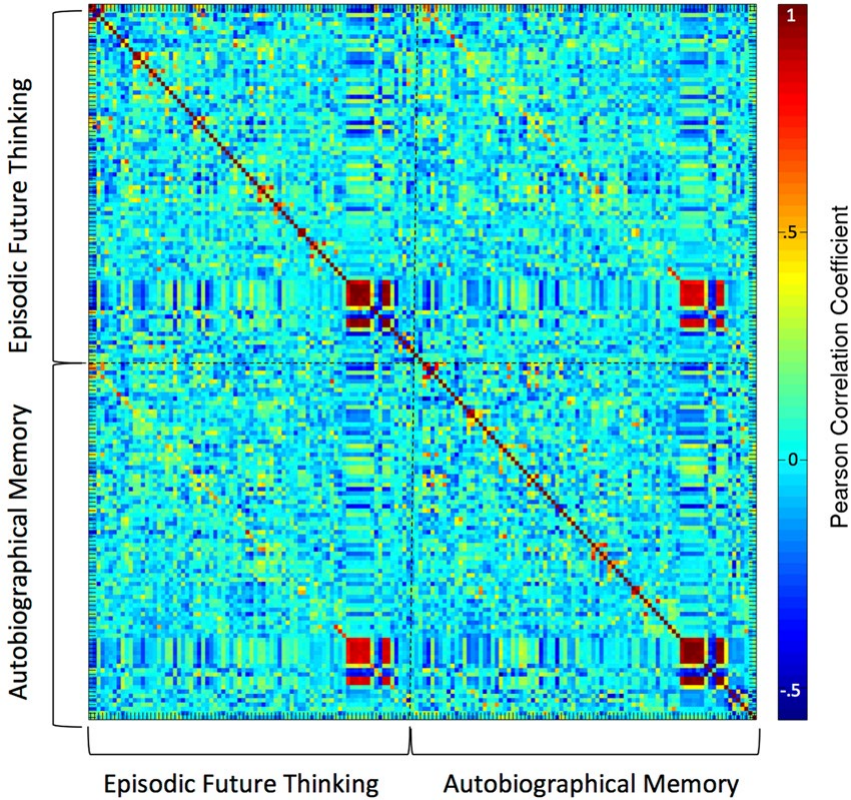
Pearson correlation matrix of all predictor variables, separated according to experimental condition (Autobiographical Memory [AM], Episodic Future Thinking [EFT]). See Fig. 4 for a complete listing of predictor variables.

Figure 3 depicts the Pearson correlation matrix of the entire set of 166 predictor variables (19 manual features + 64 LIWC features per condition). Substantial collinearity between some variables is evident, in particular between the different variables measuring text quantity. Also, many measures showed considerable consistency across the two conditions (note the diagonal in the lower quadrant of the correlation matrix with generally positive correlation values). These correlations were highest for variables related to material quantity (number of words, number of sentences). But also, more qualitative measures such as the degree to which particular word classes were used (e.g. *proportion of adjectives/verbs/nouns*) as well as *words per sentence* and average BAWL imageability ratings were positively correlated between conditions. Together, these data suggest that both quantitative and qualitative aspects of the narratives were correlated between conditions. The correlation between manually derived text features and LIWC features was generally quite low, suggesting that the two types of features were not redundant.

Interestingly, *proportion of adjectives* was positively correlated with most measures of raw material quantity, suggesting that the proportion of adjectives tended to increase with increasing lengths of the narratives - subjects producing longer narratives also incorporated a relatively greater number of adjectives in those narratives. Similarly, *words per sentence* was positively correlated with most measures of quantity, such that subjects producing longer narratives also produced relatively longer (and potentially more complex) sentences.

### Principal component analysis and regression

**Figure 4:**
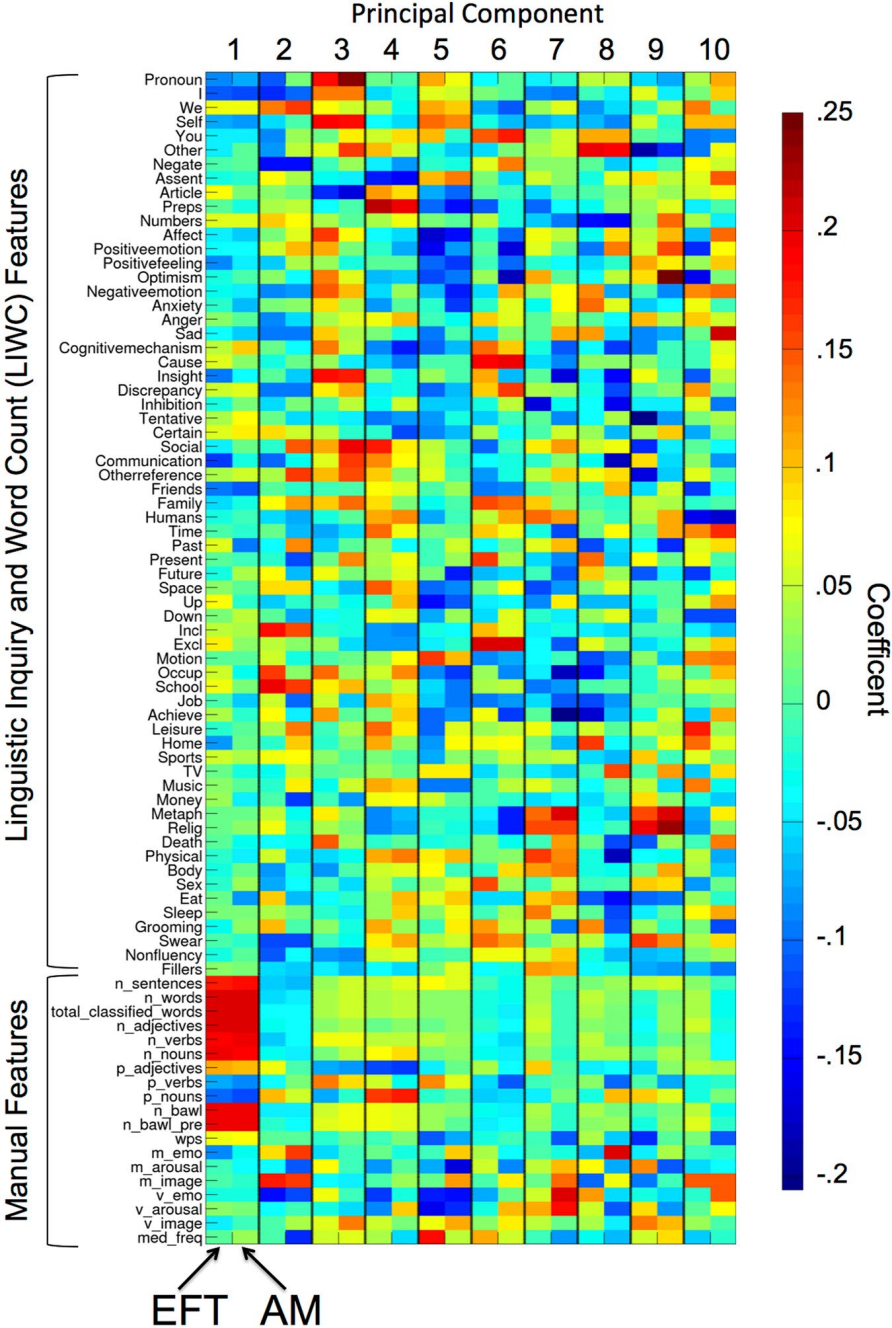
PCA coefficients per predictor and experimental condition for the first ten principal components of the data matrix plotted in Fig. 3. Note that coefficients for EFT and AM conditions are plotted next to each other to illustrate the similarity in loadings across conditions for the first few components. Note that “manual features” refer to those text features that were extracted manually (see Table 1 for details). “LIWC features” are those text features that were extracted using a German version of the Linguistic Inquiry and Word Count Software^31^.

We next performed a principal component analysis (PCA) on the data to address the multi-collinearity problem (see Fig. 1). Figure 4 illustrates predictor loadings of the first 10 principal components, and also illustrates the loading similarity between conditions. Similarity between conditions was most pronounced for the first few components, and decreased with increasing component number.

**Figure 5:**
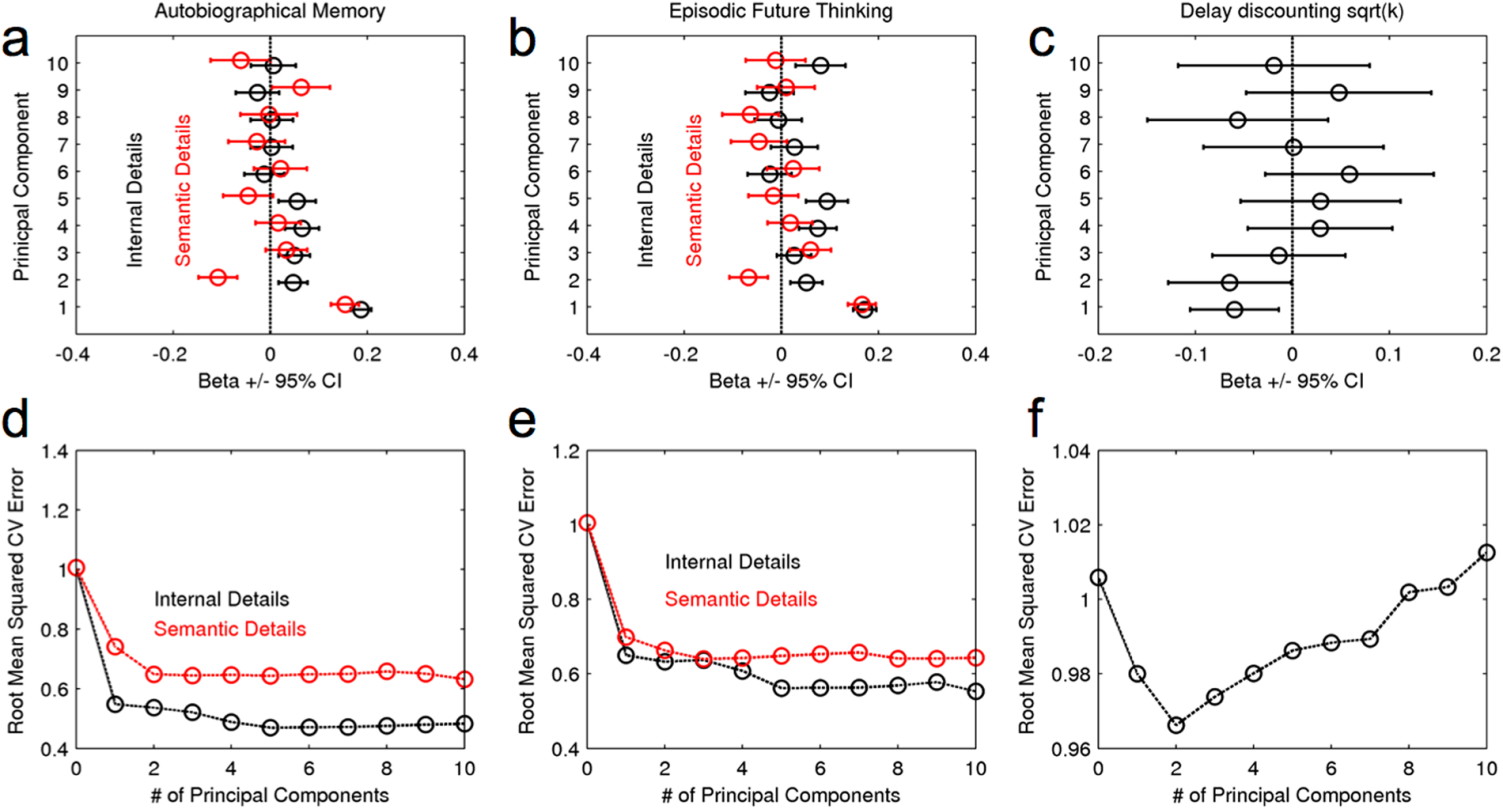
Regression coefficients (+/− 95% confidence intervals) from a regression of internal (black) and semantic details ratings (red) onto the first ten principal components of text features (see Fig. 4).

We next used principal component regression (PCR) to predict different measures using the extracted text features. In particular, we set up five analogous PCR models, all of which used the entire data matrix **X** for prediction. Model 1 and 2 predicted AM internal (episodic) and semantic details sum scores, respectively, models 3 and 4 predicted EFT internal and semantic details sum scores, and model 5 predicted square-root-transformed discount rates. As the PCA components used for prediction are solely based on the data, they were identical across models. This approach enabled us therefore to assess whether e.g. internal vs. semantic details scores were associated with different text features. Figure 5 (top row) shows the regression coefficients for the first 10 PCs for each model. Internal and semantic details for AM and EFT were all positively and significantly (i.e. the 95% CI did not include 0) associated with the 1^st^ PC (which predominantly reflects text quantity, see Fig. 4). In contrast, e.g. the 2^nd^ and 5^th^ components (both reflecting more qualitative aspects of the narratives, see Fig. 4) were significantly positively associated with internal but not semantic details for both AM and EFT. PCR models with 5 components also produced the lowest cross validation error, i.e. best out-of-sample prediction (Fig. 5).

Interestingly, the 1^st^ and 2^nd^ PCs also showed a significant negative association with square-root transformed discount rates (95% CIs for both regression coefficients were <0). Out of sample prediction, again estimated using leave-one-out cross-validation, revealed lowest prediction error for the two-component PCR model. However, note that overall prediction accuracy of temporal discounting was only marginally better than the 0-component (i.e. intercept only) model.

### Alternative regression approaches

**Figure 6:**
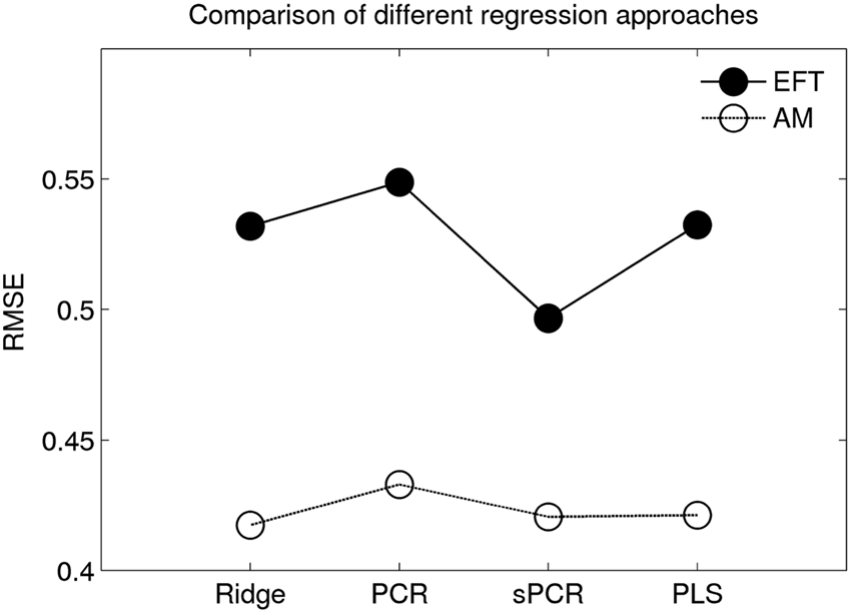
Comparison of regression techniques. Plotted are cross-validation errors (root mean squared error, RMSE) for four different techniques (Ridge – Ridge regression, PCR – Principal Component Regression, sPCR – supervised principal component regression, PLS – Partial Least Squares), predicting internal details ratings for EFT (black circles, solid lines) and AM (white circles, dashed lines). Prediction accuracy was very similar for the different approaches.

**Figure 7:**
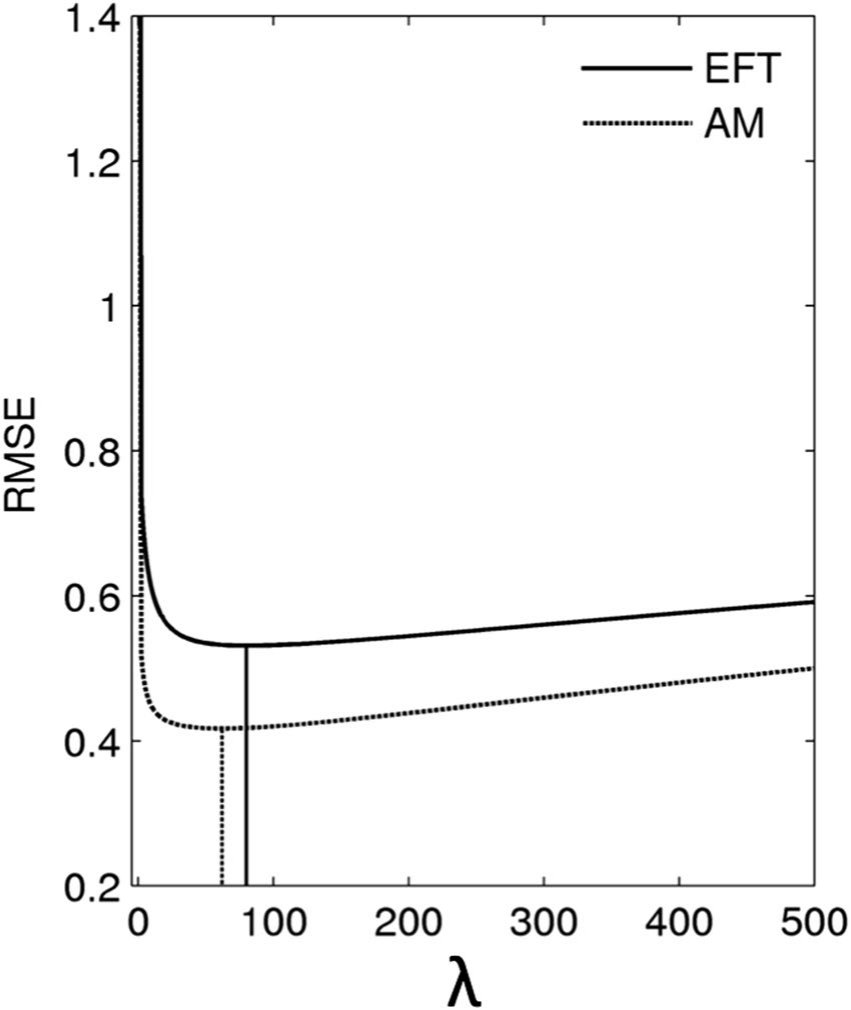
Cross-validation results for the ridge regression models. Cross-validation errors (root mean square error, RMSE) for EFT (solid line) and AM (dashed line) are plotted as a function of the ridge shrinkage parameter $\lambda$. Values of $\lambda$ yielding the lowest RMSE are marked using vertical lines.

For comparison, we repeated the prediction analyses of AI details sum scores using 1) Ridge regression, 2) supervised PCR and 3) PLS (see methods section for details). Best fitting hyper-parameters for these models (e.g. shrinkage parameter $\lambda$ for ridge regression, cut-off parameter $\theta$ for sPCR) where again determined by LOO cross-validation, and are listed in Table 2. As expected, absolute differences between the regression approaches were small^32^. RMSE for the best fitting model of each class is plotted in Fig. 6. For the ridge regression model, Fig. 7 plots $\lambda$ against the cross-validation error (RMSE).

**Table 2:** Hyper-parameters of the best-fitting regression models as determined by leave-one-out cross-validation (*n* – number of components included in the model, $\lambda$ – ridge shrinkage parameter, $\theta$ – sPCR cut-off parameter).

	PCR	Ridge	Supervised PCR			PLS:
	*n*	*λ*	*θ*		*n*	*n*
EFT_internal details_	5	80	46		9	2
AM_internal details_	5	62	51		4	3
Discounting sqrt(k)	2	1168	51		4	1

### Comparison of LIWC and manual features

**Figure 8:**
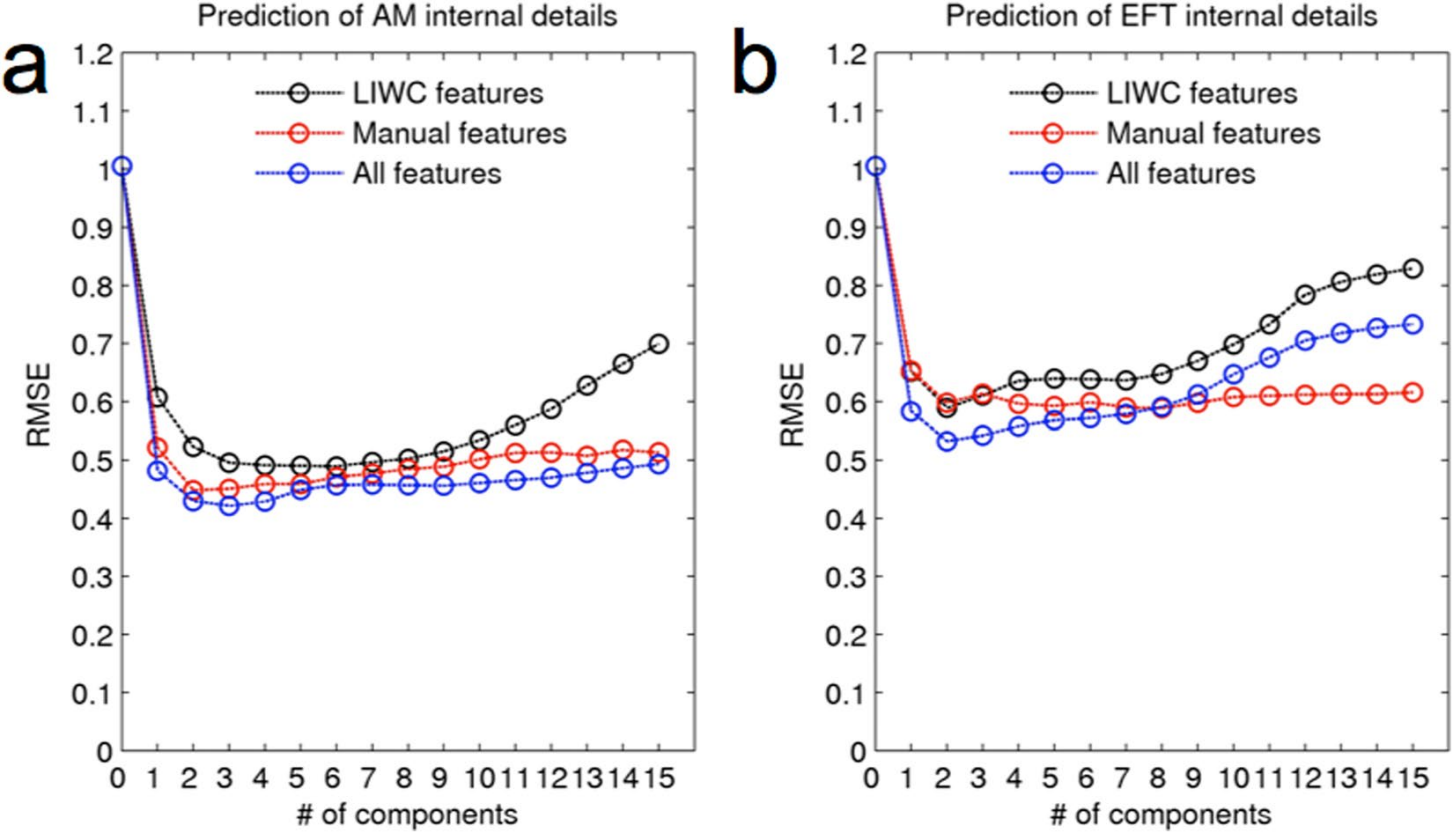
Comparison of the effects of training with different classes of features on cross-validation errors (root mean square error, RMSE) for prediction of AM internal details (**a**) and EFT internal details (**b**) using partial least squares (PLS). black: only linguistic inquiry and word count (LIWC) features used for training, red: only manual features used for training, blue: all features used for training. See methods section for details on these different feature sets. RMSE was lowest when using all features for prediction, and highest when using only LIWC features.

We then examined the added value of LIWC vs. manual features in predicting internal detail sum scores. To this end, we compared PLS models that were trained on either all features, only LIWC features or only manual features, and compared the prediction accuracies in terms of RMSE. As can be seen from Fig. 8, prediction using the combined feature set was better than prediction using either feature set alone, as indexed by a lower cross-validation error. This was the case for both prediction of AM internal details scores (Fig. 8a) and EFT internal details scores (Fig. 8b).

### Effects of the size of the test data set

Finally, we explored how reducing the amount of available test data affects prediction accuracy. To this end, we systematically varied the amount of test data used for prediction (but not the amount of training data). For simplicity, the following analyses used only the manual text features, and not the LIWC features. Given the similar results for the different regression approaches in the previous sections, we focussed on 2-component PLS models. Also, we used AM data for predicting AM ratings and EFT data to predict EFT ratings. Thus, for EFT and AM each, a separate 2-component PLS model trained on the respective feature sets and was used for prediction. We then varied the amount of test data used for prediction. Note that this resembles a situation where one has a model trained on a large data set, but would like to more efficiently predict AM/EFT in a subsequent shorter test session.

**Figure 9:**
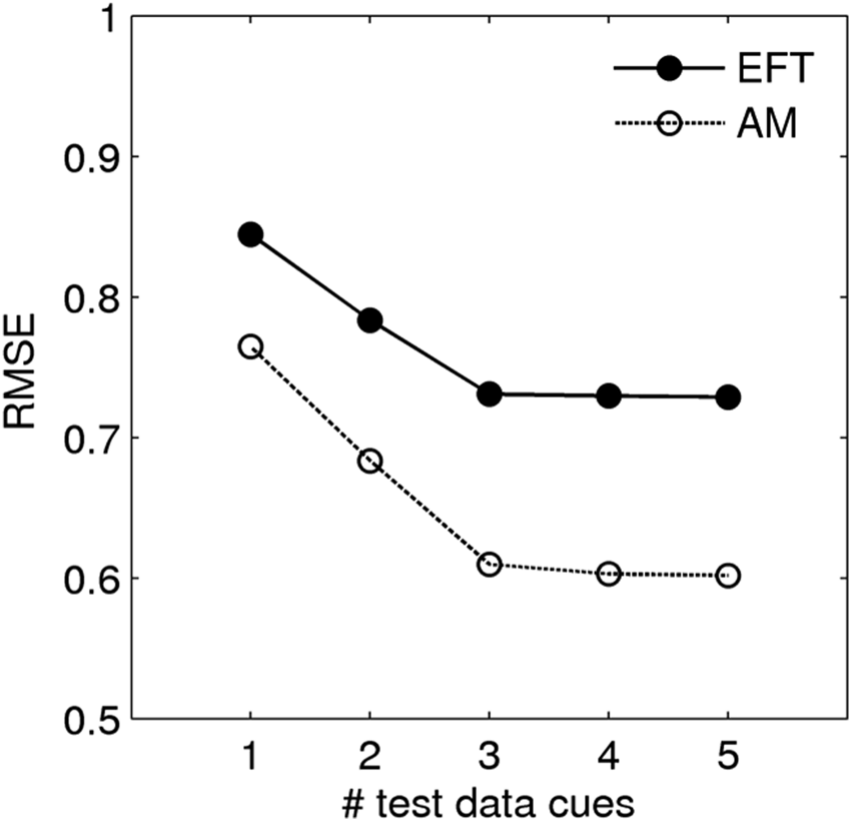
Effects of varying the amount of test data on prediction accuracy of a 2-component partial least squares (PLS) model. The X-axis depicts the number of event cues in the test data (see methods section). The Y-axis depicts the cross-validation error (root mean square error, RMSE). Solid line: prediction of EFT internal details EFT. Dashed line: prediction of AM internal details. Note that this analysis used the EFT text features to predict EFT details scores, and AM text features to predict AM details scores and that only manual features and not LIWC features (see methods) were used.

We first computed all manual features (see Table 1) separately for each cue (see section “Autobiographical Interview” in the methods section). Second, we fit 2-component PLS models to the data from all but one subject (leave-one-out cross-validation, see methods section). However, unlike the previous analyses, were we averaged the root mean squared cross-validation error (RMSE) across subjects (see methods), we now first averaged the cross-validation error across all possible subsets of test data of a given size. For each subject and test data size (i.e. 1 to 5 event cues), we averaged the cross-validation error across all possible test data subsets, and then computed the RMSE across subjects. The result is plotted in Fig. 9: the RMSE decreased from 1 to 3 cues, but for n ≥ 3 event cues, no further improvement in prediction was observed. This was the case for both AM and EFT data. Note that overall, prediction accuracy was lower than in the previous models, because in this analysis only condition-specific data were used for prediction.

## Discussion

The ability to remember the past and to project oneself into the future is a core human cognitive capacity that is impaired in a range of psychiatric and neurological disorders. Memory and prospection processes are typically measured using variations of the Autobiographical Interview (AI), a procedure that combines verbal event elaboration with a manual rating procedure to quantify the episodic and semantic content of the narratives^20^. Here we explore for the first time methods to analyze AI data using automatic extraction of low-level linguistic features. In a first proof-of-concept approach, we applied regression techniques to predict standard AI details sum scores for internal (i.e. episodic) and semantic details from these low-level text features. Our findings suggest that AI detail sum scores can be predicted with reasonable accuracy from basic linguistic text features, with prediction accuracy averaging at about 0.5 standard deviations across analyses. Additional analyses show that different linguistic text features are associated with episodic and semantic information.

First, our analyses of a relatively large number of AI interviews (n = 86) show that subject’s event elaborations for past (AM) and future events (EFT) are correlated, not only in terms of the detail sum scores, but also in terms of lower-level text features: both quantitative (e.g. words per sentence, total number of words) and qualitative characteristics of the narratives (e.g. proportion of adjectives, mean word imageability) were significantly correlated between conditions. In particular, the significant correlations of measures such as average word imageability ratings or word class proportions suggest that these features might capture meaningful between-subject variability. The results from principal component regression models used to predict internal and semantic details sum scores from AM and EFT revealed very similar patterns for the two experimental conditions. Taken together, these results support previous findings of a close association between AM and EFT^1^.

Second, results from the PCR show that generally, both episodic (internal) and semantic details scores from the AI are strongly correlated with the raw quantity of verbal material produced by participants (first principal component). PCR revealed additional more qualitative components (e.g. the 2^nd^ principal component with high loadings on word imageability and valence) that where associated with episodic rather than semantic detail ratings. That is, use of more positive-valenced words and highly imageable words was correlated with greater episodic but not semantic details sum scores. Taken together, these findings suggest that AI sum scores do not solely capture variance due to narrative quantity, but also variance due to the quality of the elaborations. This supports previously reported dissociations between episodic and semantic memory and future thinking in different patient groups and age groups that have typically not reported group differences in narrative quantity^2–4,34^. We show that although narrative quantity is associated with both internal and semantic detail sum scores, narrative quality, as measured by linguistic text features, independently contributes to variability in these AI sum scores.

We compared a number of different regression approaches in terms of prediction accuracy. These analyses confirmed that the performance of the different regression techniques was very similar. Of note, the relatively new approach of supervised principal component regression (sPCR)^33^ performed quite well in particular for the EFT condition, which might be of interest for future studies.

In the light of the known association between EFT and temporal discounting^12,14–16,18,22,35^, we also explored the extend to which discounting behavior can be directly predicted from AI text features. The first two principal components from the text feature data were significantly associated with square-root-transformed discount rates (i.e. the 95% confidence intervals did not include 0). This is of interest, since these are the same components that also showed an association with internal (i.e. episodic) details. Yet, for prediction of discount rates, the out-of-sample prediction accuracy of the best 2-component PCR model was low and only marginally better than an intercept-only model. Despite the square-root-transformation, the distribution of discount rates was still somewhat skewed, and this association may in part be driven by a relatively small number of participants with relatively high discount rates. We previously reported a reliable association between discounting and EFT internal details scores in the adolescent subsample of the present data set^22^. Although more data are clearly required, together, these findings suggest that temporal discounting might be more directly related to AI details scores than the text feature data examined in the present study.

We also assessed the added value of LIWC features and manually computed word features in prediction of AI data. Our findings suggest that the use of a combination of dictionary-based methods such as the LIWC and word-feature methods (e.g. the manual feature extraction methods employed here) may yield better prediction accuracy for AI detail scores than either feature class alone. Future studies on quantitative text analysis might benefit from complementing dictionary-based methods such as the LIWC with additional text features such as those examined in the present study (e.g. proportions of different word classes; imageability, valence and arousal scores, etc.).

Finally, by systematically varying the size of the test data sets, we could show that increasing the test data size beyond n = 3 event cues per participant and condition may not further improve prediction accuracy. This might be of interest for future studies employing the methods described here for a semi-automatic analysis of novel AI data sets. There may be an upper limit for our feature-based prediction approach that is reached with considerably smaller test data sizes than typically used in studies employing the AI.

We acknowledge that attempts were made in the context of the AI to differentiate between different types of content of the narratives. Internal details sum scores are derived from separate, theory-driven detail counts pertaining to perceptual, emotional, spatial, temporal or event information^20^. However, for the present study we focused on the most widely used outcome measures of the AI (internal vs. semantic details), partly because the variance in some of the more specific detail categories tends to be quite low. This makes these more specific outcome variables less suitable for between-subject prediction. However, it would be interesting for future studies to explore the degree to which the different subtypes of internal details map onto different linguistic profiles.

One important limitation of the present approach (and of quantitative text analysis approaches such as LIWC in general) is that these approaches focus solely on word-level information. That is, semantic differences between sentences such as “I felt very stressed that day” (internal detail) and “I always feel very stressed” (semantic detail) are ignored by such automatic approaches. The same holds for the difference between internal and external details, which during AI scoring depends on semantics. In our approach, these different types of AI details can in principle only be dissociated indirectly via a differential association with lower-level linguistic features, but never based on sentence semantics. It is likely that this exclusive use of word-level information leads to the prediction accuracy bounding at around 0.5 standard deviations.

Although the vast majority of studies using the AI are conducted in English speaking subjects, we analyzed German AI data. A validated LIWC dictionary was used^31^ and measures such as valence, arousal and imageability were extracted from a large published German word data base^36^. A translation of the present approach to English language AI data would likely be of considerable interest, but also require additional programming efforts.

The present analyses constitute one of the first steps towards a more automatic analysis of AI data^25^. We focused on exploring the association between the commonly used AI details sum scores and automatically extracted text features. A number interesting research questions remain that were beyond the scope of this initial proof-of-concept report. First, we pooled data across three very different subject groups covering a considerable age range. Exploring how linguistic features vary as a function of factors such as age^25^, clinical status^26,27^ or a range of other psychological constructs would be of considerable interest. Second, a comprehensive comparison of feature scores between AM and EFT was beyond the scope of the present paper, but might reveal interesting differences in how memory and future imaginations are processed. It would also be of considerable interest to further explore how such potential differences change e.g. across the life-span^25,37^ or as a result of neurological or psychiatric disease.
